# Mapping and CRISPR homology-directed repair of a recessive white eye mutation in *Plodia* moths

**DOI:** 10.1016/j.isci.2022.103885

**Published:** 2022-02-05

**Authors:** Christa Heryanto, Joseph J. Hanly, Anyi Mazo-Vargas, Amruta Tendolkar, Arnaud Martin

**Affiliations:** 1 Department of Biological Sciences, The George Washington University, 800 22nd Street NW, Washington, DC 20052, USA

**Keywords:** Entomology, Genetic engineering, Genomics, Techniques in genetics

## Abstract

The pantry moth *Plodia interpunctella* is a worldwide pest of stored food products and a promising laboratory model system for lepidopteran functional genomics. Here we describe efficient methods for precise genome editing in this insect. A spontaneous recessive white-eyed phenotype maps to a frameshift deletion (*c*.*737delC*) in the *white* gene. CRISPR NHEJ mutagenesis of *white* replicates this phenotype with high rates of somatic biallelic knockout. G_0_ individuals with mutant clones on both eyes produced 100% mutant progeny, making *white* an ideal marker for co-conversion when targeting other genes. CRISPR HDR experiments corrected *c*.*737delC* and reverted white eyes to a pigmented state in 37% of G_0_ mosaic adults. These repaired alleles showed practical rates of germline transmission in backcrosses, demonstrating the potential of the technique for precise genome editing. *Plodia* offers a promising avenue for research in this taxon because of its lab-ready features, egg injectability, and editability.

## Highlights

• *Plodia* pantry moths are an emerging model organism for functional genomics in Lepidoptera
• Spontaneous and CRISPR-induced *white* mutations yield recessive-white eye phenotypes
• CRISPR HDR repair with ssODN donor result in practical rates of base editing
• We provide optimized protocols for *Plodia* handling and genome editing

## Graphical abstract

**Figure:**
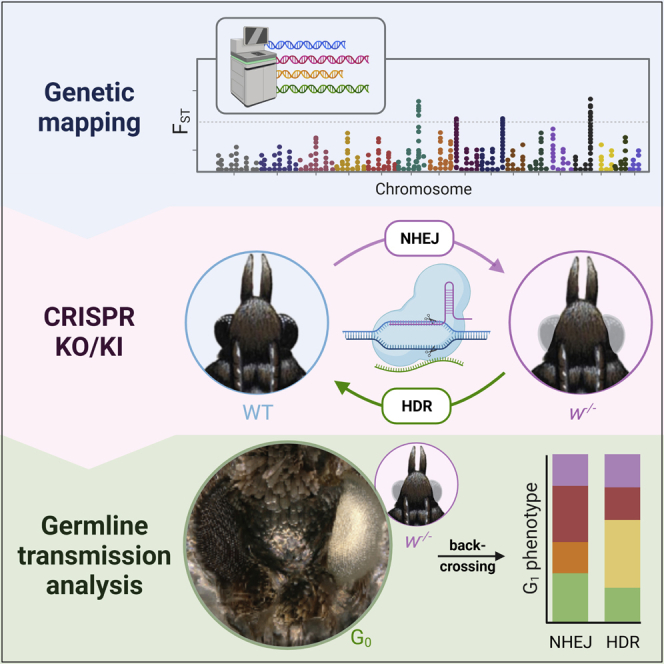

## Introduction

Emerging model organisms in functional genetics are opening new opportunities to study gene-to-phenotype relationships across the tree of life. Ideally, in addition to practical life history traits that facilitate their laboratory maintenance, animals that are tailored to genetic investigation will also be suitable for genetic transformation or editing, and harbor recessive phenotypes that can be used as selection markers for long-term line maintenance. The domesticated silkworm *Bombyx mori* fulfills these criteria and has been a flagship model organism for insect biology, with a long history of use for scientific inquiry, pioneering contributions in genomics, hundreds of mapped mutations, and rapid advances in genome editing (Goldsmith et al., 2005; Ma et al., 2019; Meng et al., 2017; Xu and O’Brochta, 2015). However, the demanding feeding, space, and handling requirements of this large-sized insect limit its widespread adoption by new laboratories interested in the biology of Lepidoptera, i.e., butterflies and moths. Lepidoptera is a large insect order that comprises 160,000 species, accounting for about 10% of all living species named to date (Kristensen et al., 2007; Beccaloni et al., 2012). As it includes important agricultural pests, ecosystem service providers, and model systems in conservation biology, ecology, and evolutionary biology, there is a growing need for developing robust and tractable model systems for functional genomics in this order.

The indianmeal moth, *Plodia interpunctella* (hereafter *Plodia; abbr. Pi*), also known as the pantry moth, is a promising emerging model system with existing genome and transcriptome assemblies (Harrison et al., 2012; Roberts et al., 2020; Tang et al., 2017) and life history traits that make it suitable for the long-term maintenance of inbred lines. Much like *Tribolium*, *Plodia* is a worldwide pest of stored food products and exhibits convenient laboratory features, including a short life cycle (25 days at 28°C), ease of culture at high density on a low-cost diet (Silhacek and Miller, 1972), inbreeding resistance (Bartlett et al., 2018), and high fecundity (Mbata, 1985). Stock keeping requires minimal human intervention because individuals enter diapause under overcrowding, short photoperiod, or low temperatures (Bell, 1994). In sharp contrast with most other lepidopteran pests, *Plodia* feeds on a dry diet rather than on fresh plant tissues, and under optimal conditions that avoid mold, there is no requirement for food replacement or waste removal over an entire life cycle, and each stock requires 15 min of work per month for basic maintenance. Egg fertilization and mass-laying are stimulated by CO_2_ narcosis, which usually generates dozens of eggs per min for immediate injection, within the time frame of the first cell divisions (Bossin et al., 2007; Dyby and Silhacek, 1997). Unmated females and males can also be isolated before adult emergence for controlled matings and crosses, allowing controlled genetic crossing. Taken together, these properties make *Plodia* a robust organism for routine germline modification, stock keeping, and isogenic or transgenic line sharing in Lepidoptera.

The *white* gene encodes a transmembrane protein of the ATP binding cassette (ABC) transporter superfamily (Mackenzie et al., 1999), with conserved functions in the transport of ommochrome and pterin pigment precursors across insects (Figon and Casas, 2019; Grubbs et al., 2015; Tatematsu et al., 2011). Because insects universally use one of these two pigment classes as filtering pigments in their compound eyes (Summers et al., 1982), *white* loss-of-function mutations generate eye color defects that are easy to screen visually (Ge et al., 2016), and played a foundational role in the early history of genetics (Green, 1996). These features have made *white* a conventional target for developing targeted mutagenesis methods in insects (Bai et al., 2019; Heu et al., 2020; Li et al., 2020; Liu et al., 2021; Ohde et al., 2018; Sim et al., 2019), though it should be noted that its knockout is recessive-lethal in a subset of tested species (Khan et al., 2017; Reding and Pick, 2020).

Recently, Shirk described an efficient method for targeted mutagenesis using CRISPR procedures that knocked out the *white* gene in *P*. *interpunctella* (Shirk, 2021). Following embryonic injections of Cas9/sgRNA duplexes targeting the coding position 242 of *white*, the author obtained G_0_ individuals with mosaic white eyes because of nonhomologous end-joining (NHEJ), resulting in biallelic knockouts caused by frameshift mutations in the *white* exon 1. They then outcrossed *Piw-*^*242*^ G_0_ mosaic females to males from the laboratory strain *Piw-*, which shows autosomal recessive inheritance of a white-eye phenotype. F_1_ offspring that inherited both a *Piw-*^*242*^ and *Piw-* were white-eyed, demonstrating the two types of alleles do not complement, and suggesting *Piw-* is a spontaneous loss-of-function mutation at the *white* locus itself. In this study, we followed up on these results with a focus on the *Piw-*spontaneous mutation, mapped this recessive marker to the region of chromosome 10 that includes the *white* gene, identified a one bp frameshift mutation in the *white* exon five causing the recessive-white phenotype, and used CRISPR editing with homology-directed repair (HDR) to rescue this mutation to a wild-type state. We also include detailed protocols for the maintenance and genome editing of *Plodia* stocks. Together, these studies enable *Plodia* as a tractable lepidopteran model organism for functional genetics via precise genome editing using the CRISPR/Cas9 system.

## Results

### Introgression of a spontaneous recessive-white marker in a genome strain

**Figure 1:**
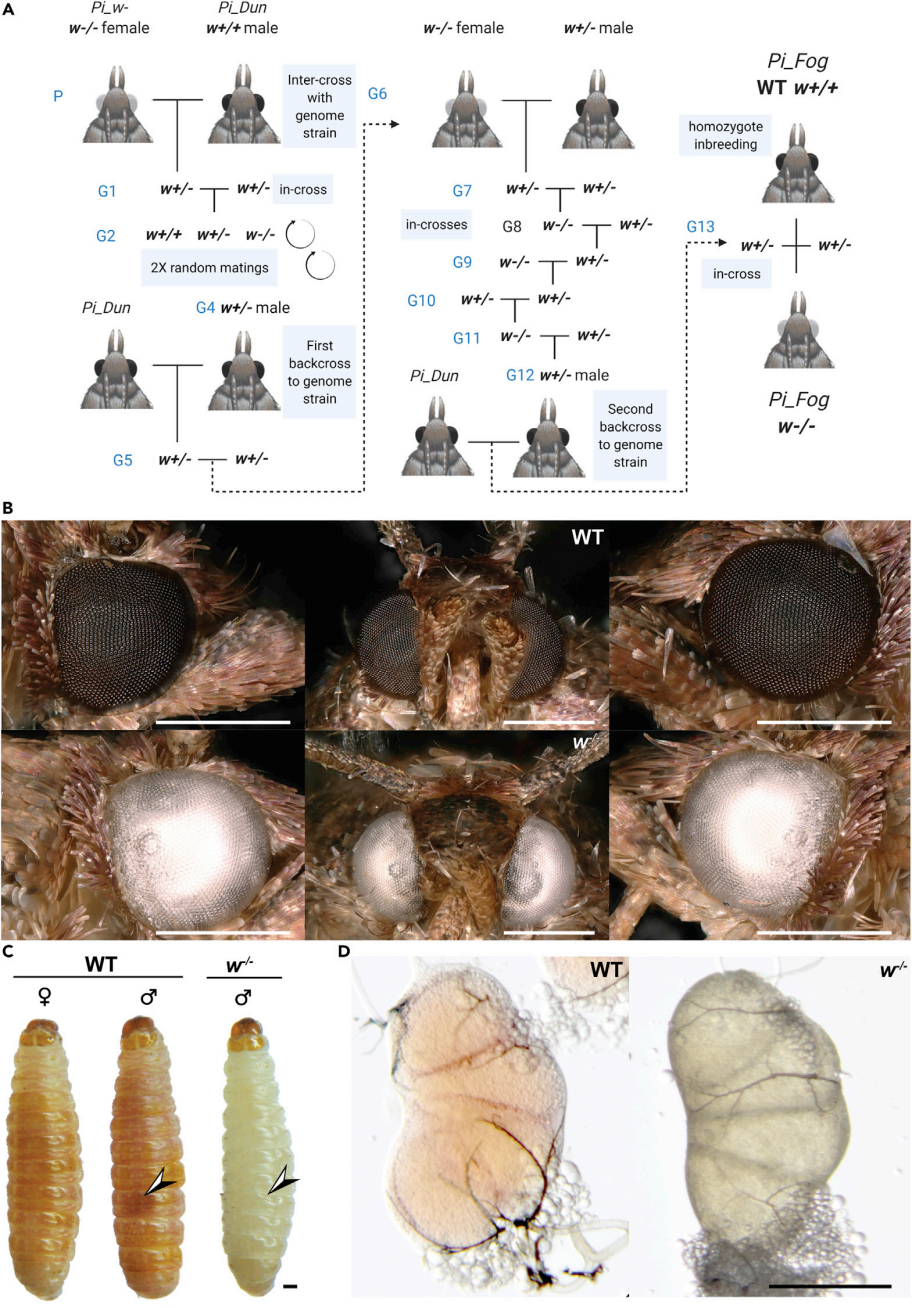
Introgression of the *w-*spontaneous mutation in a reference genome strain (A). Crossing scheme resulting in the generation of the *Pi_Fog w^-/-^* and WT (*w*^*+/+*^) inbred lines. (B) Eye phenotype of the homozygous *w^-/-^* and WT genotypes in *Pi_Fog* adult moths. (C) Larval depigmentation of *w^-/-^* fifth instar larvae. Arrowhead: larval testicular mass used for sexing in WT moth larvae, but absent at the homozygous state in *w^-/-^* males; *w^-/-^* female larvae cannot be distinguished from males based on external morphology. (D) Color differences in dissected testicular masses. Scale bars = 500 μm.

We performed crosses to identify a chromosomal location for the recessive white eye marker. We used the “Dundee” (UK) inbred strain as a wild-type background (*Pi_Dun*), to take advantage of its reference genome, available under the name *Plodia_dt* in NCBI Assembly (Roberts et al., 2020). We used the *Piw-*inbred strain (Florida, US), isolated in 1986 from a spontaneous mutation at an unknown locus (Shirk, 2021), as a donor for a recessive white-eye mutation. The *w^-^* chromosome was introgressed into the Dundee background using two backcrosses (*w*^*+/+*^ x *w*^*+/-*^) separated by five *w^-/-^* x *w*^*+/-*^ incrosses (Figure 1A), resulting in a hybrid strain we dub here “Foggy Bottom” (*abbr*. *Pi_Fog*). Because crossing-over only occurs in male meiosis in Lepidoptera (Traut et al., 2007), a heterozygous male was used in each cross to foster recombination on the *w^-^* chromosome to restrict the size of the introgressed region around the mutation, and following this introgression scheme, we inbred *Pi_Fog* wild-type (WT) and *w^-/-^* individuals separately to generate two homozygous inbred lines of each eye color phenotypes. Both phenotypic stocks have since been maintained in the lab for >20 generations without decline in viability or fertility. As in the parental *Piw^-^* strain, *Pi_Fog* homozygous individuals for the introgressed *w^-/-^* mutation differ from the WT state by completely depigmented compound eyes in adults, and a lack of reddish pigmentation in the larval cuticle (Figures 1B and 1C). The testicular masses, normally clearly visible through the cuticle of male fifth instar larvae, are also depigmented (Figures 1C and 1D).

### Mapping the *w-*mutation reveals a one bp deletion causing a frameshift of the *white* gene

**Figure 2:**
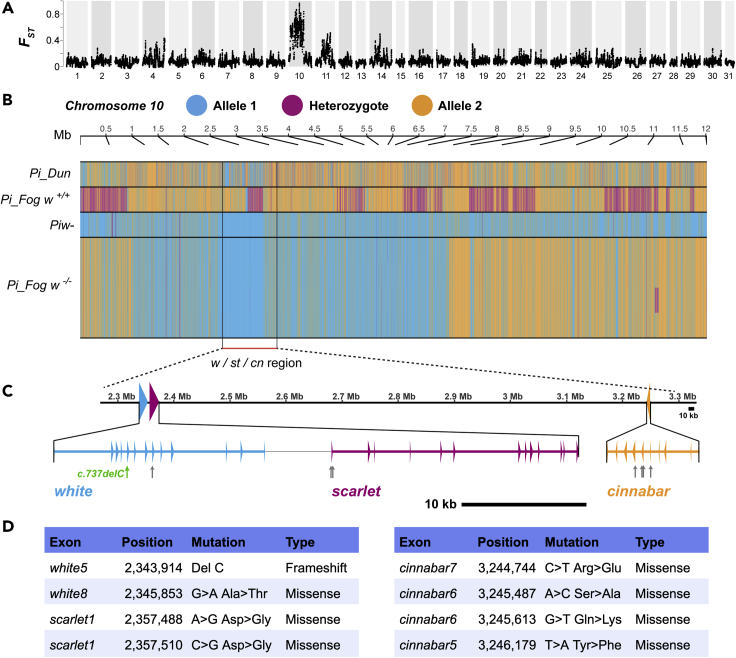
Genetic mapping of *Plodia interpunctella* eye-pigment genes within chromosome 10 (A) *F*_*ST*_ was calculated between brown-eyed individuals vs. white-eyed individuals, pinpointing one large differentiation block on chromosome 10. (B) A genotype plot of biallelic variants across chromosome 10, with the allele present in *Piw-*in blue, the alternate allele in orange, and heterozygotes in magenta, shows a large fixed block of *Piw-*haplotype in *Pi_Fog w^-/-^* individuals, centered on the *white/scarlet/cinnabar* (*w/st/cn*) locus. (C) Genetic map of *white*, *scarlet*, and *cinnabar* in chromosome 10. Arrows: coding SNPs detected in *w^-/-^* relative to WT specimens. (D) Positions of deletion and single-nucleotide polymorphisms that cause amino acid changes in three eye pigment genes. Reference states are from the WT *Pi_Fog w*^*+/+*^ individual, derived states are from resequenced recessive white-eyed *Pi_Fog w^-/-^* individuals.

In order to map the white eye mutation, we sequenced the genome of one *Piw-* individual, one brown-eyed *Pi_Fog*, and four white-eyed *Pi_Fog* individuals. We aligned the resulting reads, along with the *Pi_Dun* genome, to the chromosomal genome assembly of the US strain “Savannah” generated by the Ag100Pest consortium (Childers et al., 2021). Differentiation between the white-eyed and brown-eyed individuals as measured with *F*_*ST*_ was low except for on a portion of chromosome 10, where a large interval of differentially fixed SNPs were shared between *Piw-* and *Pi_Fog w^-/-^* individuals, but not with the *Pi_Fog w*^*+/+*^ individual or with the *Pi_Dun* individual (Figures 2A and 2B). This introgressed *w^-^* genetic interval resulted in a large haplotype that encompasses many genes, including the pigmentation gene candidates, namely *cinnabar*, *white,* and *scarlet*, a situation similar to the mapping of eye color mutants in *Bombyx* (Tatematsu et al., 2011).

**Figure 3:**
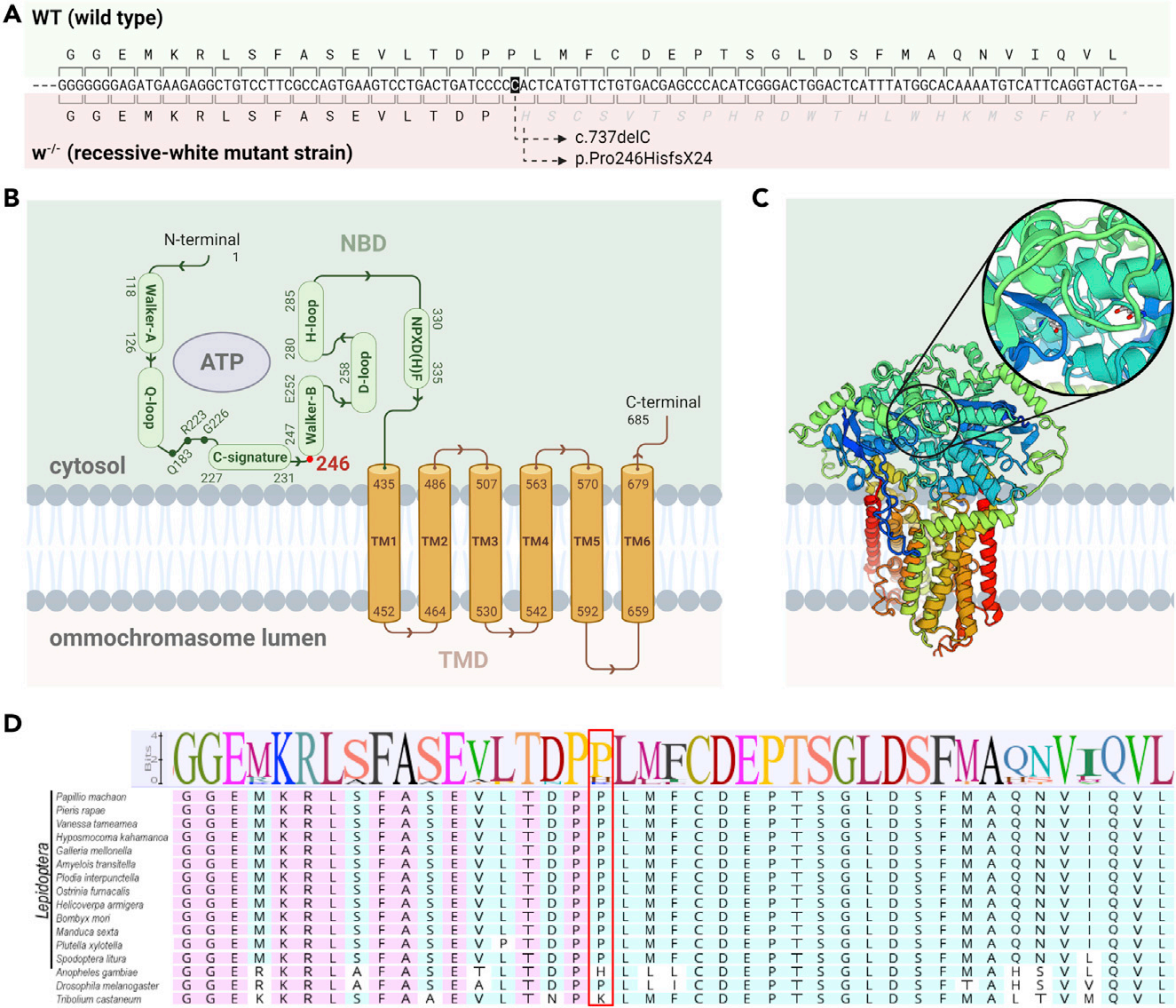
*In silico* analysis of spontaneous deletion in *Plodia interpunctella* white eye mutant (A) Cytosine deletion at position 737 in exon five of the *white* coding sequence (*c*.*737delC*, *p*.*Pro246HisfsX24*). Gray italics: frame-shifted amino acids and Stop signal (∗). (B) Half-transporter White protein motifs. Green and brown indicate the Nucleotide-Binding Domain (NBD, *syn*. ATP-binding cassette domain) and Transmembrane domain (TMD) of the protein, respectively (Eckenstaler and Benndorf, 2020). Numbers indicate amino acid positions, including the frameshift at position 246 (red). (C) Generic 3D model of White (ABCG2) half-transporter protein, shown here as a homodimer. Cooler (blue) to warmer (red) color indicates the direction from N-terminus to C-terminus. The magnified circle indicates the position of ATP binding around Glu252 residues (red dots). (D) Amino acid conservation across Lepidoptera and outgroup species around the *c*.*737delC* mutation. Red box: the conserved position where the *p*.*Pro246HisfsX24* frameshift is initiated.

We subsequently looked for coding polymorphisms in *white*, *scarlet*, and *cinnabar* loci on Chromosome 10, respectively. Aligning a *Plodia* transcriptome (Harrison et al., 2012) onto this genomic region resulted in annotation of gene structures, including a 5,953 bp *cinnabar* gene with 10 exons, a 15,893 bp *scarlet* gene with 14 exons, and a the 16,118 bp *white* gene with 13 exons (Figure 2C). Then, we examined coding SNPs in alignments of white and brown eyed sequencing reads on the chromosome 10 interval. Along with seven missense mutations (Figure 2D), we identified the *c*.*737delC* mutation in the exon five of *white* as causing a coding frameshift (*p*.*Pro246HisfsX24*) (Figure 3A). The 2,058 bp coding *white* sequence comprises 13 exons and encodes a 686 amino acid protein. The frameshift mutation is predicted to cause the loss of key components of White protein (Michalovich et al., 2002), such as all transmembrane domains, and the cytoplasmic Nucleotide Binding Domains that interact with ATP (Figures 3B and 3C). The high conservation of the amino sequence following the deletion was also illustrated through amino acid alignment among Lepidoptera and holometabolous insect outgroup species, and also highlights the functional importance of the mistranslated region following the *p*.*Pro246HisfsX24* frameshift (Figure 3D). In summary, the *c*.*737delC* mutation is null and likely causal for the recessive white phenotypes derived from the *Piw-* strain.

### CRISPR knockout of white exon five reveals efficient germline editing

Recent work described efficient CRISPR somatic mutagenesis of the *white* gene based on a sgRNA target in exon 1 (Shirk, 2021). Here, we targeted the WT allele of exon five using a sgRNA overlapping with the *c*.*737C* residue that is deleted in the *w-* strain. We aimed to measure the somatic expressivity (rate of biallelic G_0_ mutants) and germline transmission rates of CRISPR-induced mutations, effectively serving as a proxy for assessing the efficiency of NHEJ-induced modifications.

**Figure 4:**
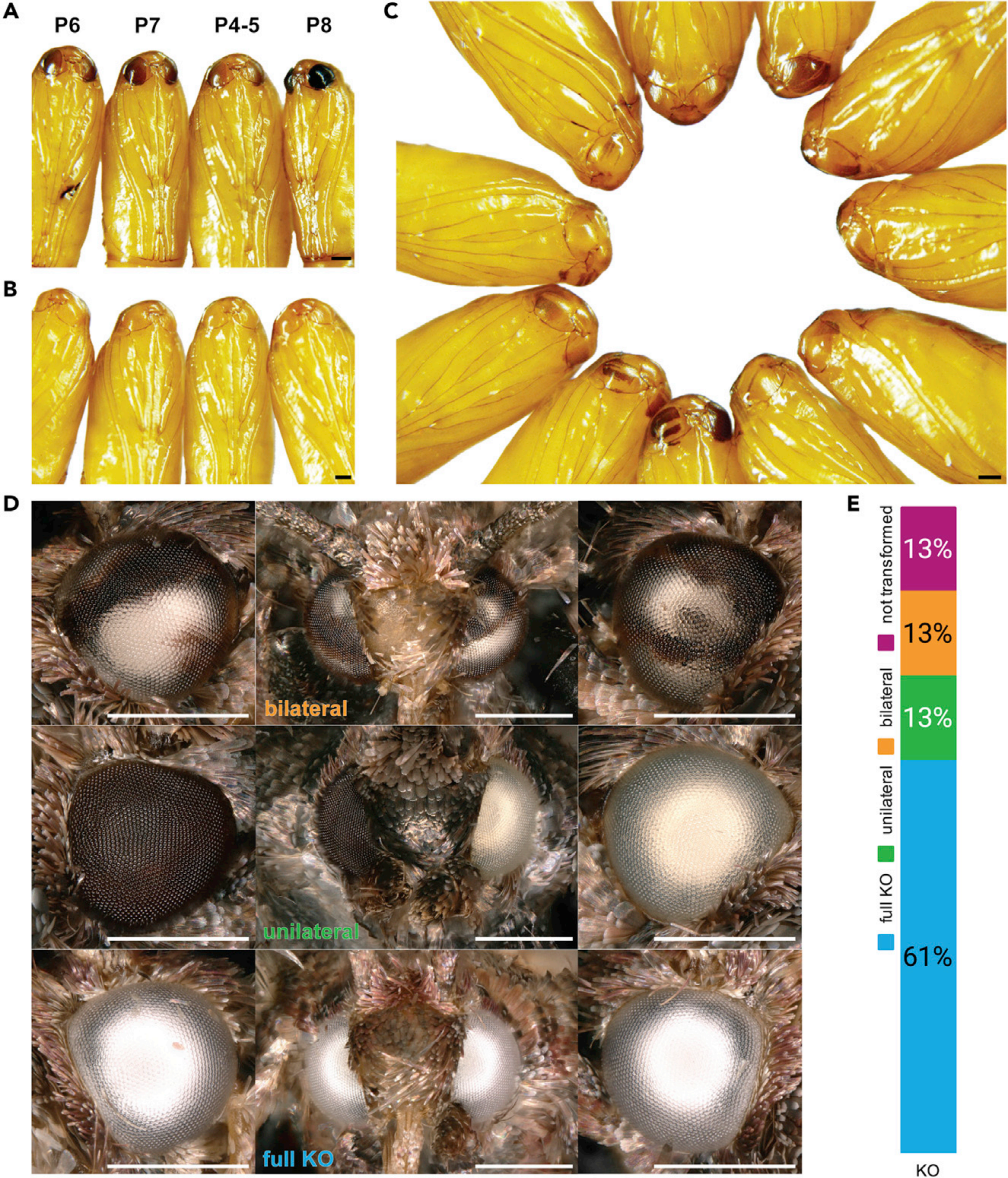
G_0_ eye color phenotypes in early pupae and adults generated by biallelic CRISPR-induced *white* mutation in wild-type *Plodia* (A) Stages of eye color progression in WT pupae. Numbers denote pupal stages (Zimowska et al., 1991). (B) Complete KO mutant phenotype in pupae, with bilateral white eyes and no change observed in eye pigmentation between P1-P9. (C) Mosaic mutant phenotypes in pupae. Eye pigmentation manifests as stripes in contrast to evenly-developing darkening from the dorsal to the ventral side of the eye in WT. (D) Mosaic mutant phenotypes in adults. Left and right columns correspond to the right and left eyes, respectively; middle columns are frontal views. Scale bars: 500 μm. (E) Phenotype proportions of knockout G_0_*Plodia* crispants.

Out of 755 WT *Plodia* eggs injected, 143 survived and grew into adulthood, of which 113 moths (79%) revealed eye color phenotypes (Figure 4 and Table 1). Mosaic phenotypes, including incomplete depigmentation of either or both eyes and unilateral absence of pigmentation, were observed in 41 G_0_ crispants (28%). Eye color development in *Plodia* pupae takes place between the P3-P9 pupal stages (Zimowska et al., 1991), and CRISPR knockout (KO) phenotypes are visible as early as P4. Interestingly, we found that upon freezing and thawing adults for imaging, mutant patches in mosaic eyes acquire a light bronze color because of pigment diffusion from adjacent wild-type clones, likely following the rupture of cell membranes in the ommatidia within 20 min after thawing (Figure S1). Complete and bilateral absence of pigmentation lacking any sign of pigment diffusion (“full KO”) was observed in 72 moths (51% of surviving adults), suggesting that the injection procedure, within 1 h after laying, can generate biallelic null mutations spanning large fractions of the soma. Together, these data highlight the high efficiency of the CRISPR procedures for generating biallelic somatic knockouts.

**Table 1:** Genome editing of wild-type *Plodia interpunctella* targeting exon five of the *white* gene

sgRNA	Injection time AEL (min)	Final concentration (ng/μL) [Cas9:sgRNA]	Total injected (N)	G_0_ Larvae	Egg hatching rate (%)	G_0_ adults (total)	G_0_ mutants (total)	G_0_ full KO mutants	G_0_ mosaic mutants
*Pi_wh5_KO_sg*	15–45	500:250	281	48	17%	46	40	28	12
*Pi_wh5_KO_sg*	19–60	500:250	474	98	21%	97	73	44	29
		**Total**	**755**	**146**	**19%**	**143**	**113**	**72**	**41**

To assess the KO efficiency in the germline, G_0_ mutants with bilateral white eyes were outcrossed to homozygous recessive mutant moths (*w*^*-/-*^). Fifteen out of 16 attempted crosses were fertile and produced a total of 1,177 white-eyed G_1_ progeny (100%), without any brown-eyed progeny (Table 2). This shows that G_0_ crispants with little somatic mosaicism, as inferred from bilateral mutant phenotypes, indeed carried 100% loss-of-function alleles in their germline. These data thus indicate the high efficiency of CRISPR procedures for generating biallelic knockouts, and that the production of germline mutations can be greatly facilitated by selecting founders in which mosaicism is minimal.

**Table 2:** Germline transmission of CRISPR-induced KO mutations following outcross of bilateral G_0_ crispants to *w*^*-/-*^

Family	G_0_ NHEJ parent with bilateral white clones	Brown G_1_*w*^*-/+*^	White G_1_ *w*^*-/NHEJ(*^^-^^*)*^	% *w*^*NHEJ(*^^*-*^^*)*^ allele transmission
1	female	0	88	100
2	female	0	65	100
3	female	0	86	100
4	male	0	89	100
5	male	0	72	100
6	male	0	95	100
7	male	0	66	100
8	male	0	38	100
9	male	0	107	100
10	male	0	74	100
11	male	0	70	10
12	male	0	88	100
13	male	0	61	100
14	male	0	83	100
15	male	0	95	100
	Total	**0**	**1177**	**100**

### Homology-directed repair mediates efficient rescue of the one bp *white* deletion

**Figure 5:**
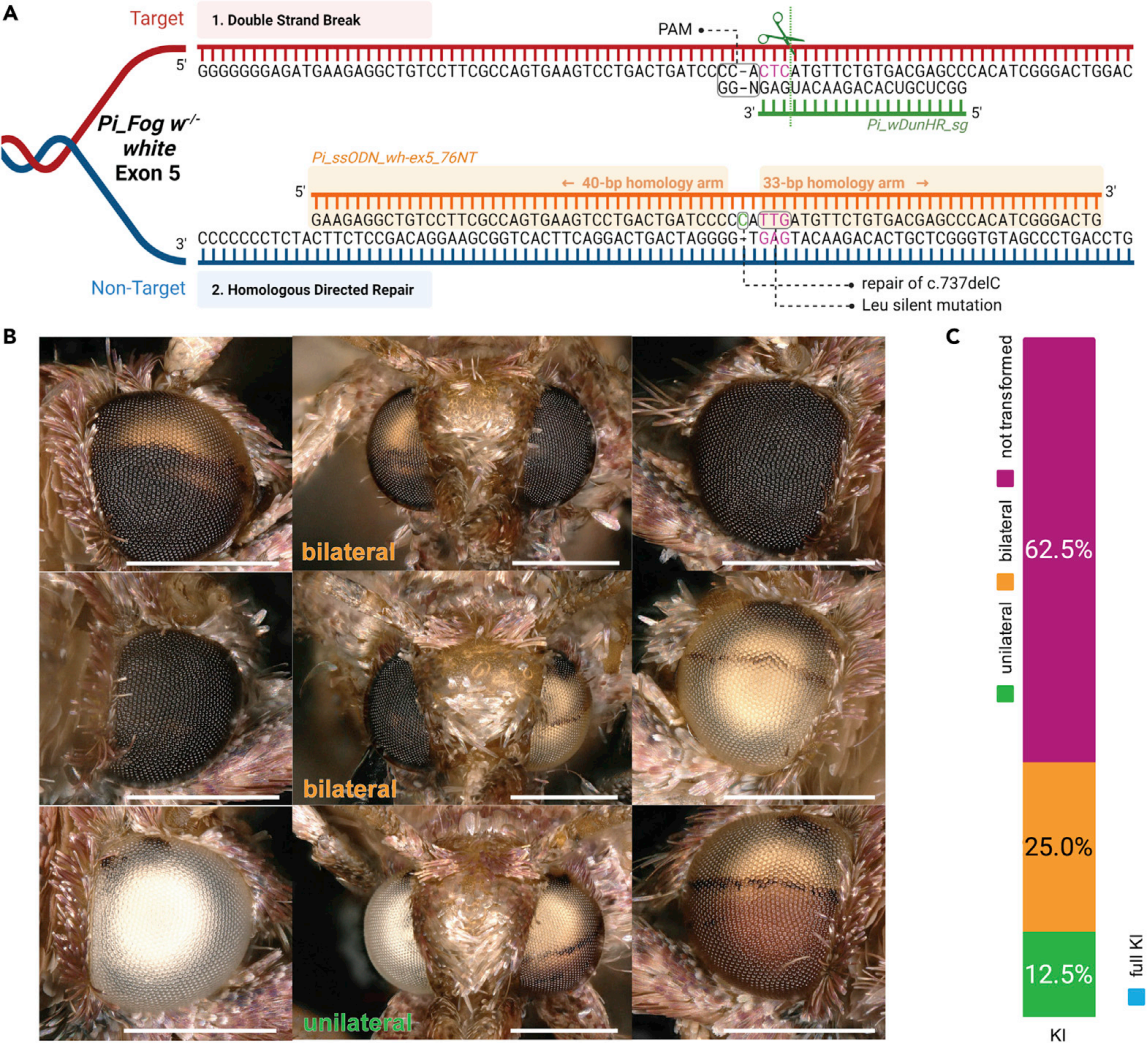
G_0_ eye color reversions generated by HDR-induced *white* rescue in white-eyed *Plodia* (A) Knock-in strategy using CRISPR Cas9/sgRNA complex and ssODN template. Green dotted line indicates the cutting site. Letters in magenta indicate the codons changed to introduce Leucine silent mutation provided by repair template *Pi_ssODN_wh-ex5_76NT*. (B) Mosaic repaired wild-type phenotypes. Bronze color is an effect of the freeze-thawing step prior to mounting for imaging (Figure S1). Scale bars : 500 μm. (C) Phenotype proportions of knock-in G_0_*Plodia* crispants.

To test the potential of precise CRISPR genome editing and HDR in *Plodia*, we sought to rescue the one bp deletion at *c*.*737delC*, based on the assumption that it is the only variant causing the white eye phenotype in the spontaneous *w*^*-/-*^ strains. We predicted that repaired alleles would be dominant, allowing a rapid quantification of the efficiency of the technique. We used a single-stranded oligodeoxynucleotide (ssODN) as a repair donor template as employed in other organisms (Gratz et al., 2013; Okamoto et al., 2019). The sgRNA and ssODN were designed such that the double-strand break (DSB) site is close to the intended edit (Figure 5A), with the ssODN made antiparallel to the nontarget (NT) strand. In addition, the ssODN was asymmetric with 40 nt and 33 nt 5′ and 3′ homology arms, flanked the intended C insertion at *c*.*737delC*, and incorporated a silent mutation preventing secondary cutting of repaired alleles. Coinjecting this donor with Cas9:sgRNA heteroduplex targeting the *c*.*737delC* allele in *w*^*-/-*^ homozygotes resulted in efficient dominant rescues of the white-eye phenotype (Tables 3 and 4). Out of 1,014 eggs injected, a total of 120 adult moths emerged, from which 45 moths (38%) exhibited a mosaic phenotype consisting of often large, brown pigmented eye clones on one or both eyes (Figure 5B). To assess the rate of germline transformation of corrected alleles, we backcrossed G_0_ mosaic mutants to the homozygous *w*^*-/-*^ stock. Because the WT allele is dominant, the proportion of G_1_ progeny with WT eye color reflects the proportion of corrected WT alleles in the G_0_ germline, which gives an estimate of the germline transformation efficiency using our CRISPR HDR approach. Ten out of 15 attempted crosses produced progenies. Out of these 10 crosses, five produced G_1_ WT individuals at ratios of 11.5–77.3% in each progeny. Sanger sequencing followed by chromatogram parsing of the heterozygous signal (Hill et al., 2014) confirmed the presence of a *w^-^* allele and of an HDR-repaired allele in these samples, with the C insertion and the two synonymous nucleotide changes intended to prevent secondary cuts.

**Table 3:** Genome editing and homology-directed repair of spontaneous *white* mutant of *Plodia interpunctella*

sgRNA	Injection time AEL (min)	Final concentration (ng/μL) [Cas9:sgRNA:ssODN]	Total injected (N)	G_0_ Larvae	Egg hatching rate (%)	G_0_ adults (total)	G_0_ with mosaic eye color rescues (total)
*Pi_wh5_HR_sg + ssODN_76NT*	15–60	500:250:1000	505	113	22.4%	64	25
*Pi_wh5_HR_sg + ssODN_76NT*	12–56	500:250:1000	509	70	13.7%	56	20
		**Total**	**1014**	**183**	**18.0%**	**120**	**45**

**Table 4:** Germline transmission of CRISPR-induced KI mutations following outcross of unilateral and bilateral G_0_ crispants to *w*^*−/*−^

Family	G_0_ HDR parent with brown clones	White G_1_ *w*^-^^*/*^^*-*^	Brown G_1_ *w*^*-/HDR(+)*^	% *w*^*HDR(+)*^ allele transmission
1	male	23	60	72.3
2	female	32	10	23.8
3	male	58	38	39.6
4	female	4	6	60.0
5	male	38	0	0.0
6	female	40	0	0.0
7	female	38	0	0.0
8	male	54	7	11.5
9	male	20	0	0.0
10	male	68	0	0.0
	Total	**375**	**121**	**32.3**

### Knockouts of the sex-linked gene *Blos2* are deleterious

Although we expect the *white* autosomal recessive marker to be convenient for many genetic manipulation experiments in the future, we also explored the possibility to generate a sex-linked mutation with a phenotypic effect that is convenient to identify. To do this, we used CRISPR NHEJ targeting the gene *Biogenesis of lysosome-related organelles complex one subunit 2* (*Blos2*). *Blos2* is located on the lepidopteran Z sex chromosome and mediates the formation of pigment-containing organelles, with viable knockout alleles generating translucent larval epidermis in several moth species (Cheli et al., 2010; Fujii et al., 2010; Lee et al., 2018; Nakade et al., 2014; Zhu et al., 2020). CRISPR mutagenesis induced G_0_ mosaic phenotypes in the larval integument, with mutant portions lacking the characteristic pink pigmentation observed in WT individuals (Figure S3A). Surviving pupae showed transient depigmentation clones in the eye before melanization (Figure S3B), at a stage where eyes are normally brown (Zimowska et al., 1991). This phenotype was not visible past 60% of pupal development (P8 stage) because of the successful deposition of a late dark melanin pigment, suggesting that *Blos2* is only necessary for the accumulation of an early eye pigment that is deposited at the mid-pupal stages (20–50% pupal development). The G_0_ egg hatching rate of 14% (203 hatchlings out of 1441 egg injections) was comparable to *white* KO injections (19%). However, we observed a low survival of the *Blos2* G_0_ mutant larvae, with only 38% (77/203) of larvae reaching the pupal stage, compared to survival rates above 98% in *white* G_0_ mutants or in uninjected stock in identical conditions. G_0_ mutants with the largest pupal eye mutant clones failed to hatch as adults, and as a matter of fact, we failed to obtain G_1_ phenotypes from several incrosses, possibly because the parents that survived did not carry mutant alleles in their germline. From these observations, we extrapolate that *Blos2* null mutations are deleterious in *Plodia*, and thus that they are not a viable phenotypic marker of the Z chromosome over several generations.

## Discussion

Pigmentation loci have been universally used in insects as a phenotypic marker for various molecular manipulations (Bai et al., 2019; Khan et al., 2017; Xu et al., 2020) because of their easily distinguishable body color at the larval stage and eye color at the adult stage. In the first effort to knockout eye color genes in *Plodia* using CRISPR/Cas9 study (Shirk, 2021), the first exon of the *white* locus was targeted to produce a heritable null mutation. Here, we investigated the genetic basis of a spontaneous eye color mutant phenotype in *Plodia* by whole-genome sequencing and demonstrated a successful workflow for functional study through precise genome editing. Specifically, CRISPR-mediated NHEJ knockout of *white* resulted in both biallelic somatic editing and germline transmission at relatively high efficiency. Last, we showed that HDR knock-in with a ssODN donor efficiently corrected a one bp deletion causing recessive white eyes, again with high efficiency. These data, together with streamlined and safe rearing procedures, lay the foundation for further use of *Plodia* in functional genomics.

We propose that the depigmentation of white-eyed *Pi_Fog w*^*-/-*^ makes it ideal for the addition of dominant markers, such as fluorescent proteins driven by the *3xP3* eye and glia promoter that is known to work in *Plodia* (Bossin et al., 2007). The wild-type *Pi_Fog* could be used for CRISPR experiments where the *white* knockout sgRNA is coinjected to select founders with large clones, a co-conversion strategy already used in *Drosophila* (Ge et al., 2016). Indeed, our knockout data indicated that 100% (N = 15) of G_0_ founders with bilateral, non-mosaic white eyes because of biallelic KO, generated 100% offspring (N = 1,177). In other words, white eye phenotypes provide a suitable marker for identifying founders that carry germline edits, and we suggest this co-conversion strategy will be instrumental for establishing stable genome edited lines where it is difficult to screen for phenotypes. The *Pi_Dun* genome reference is readily accessible online on the NCBI Assembly server under the name *Pi_dt* (Roberts et al., 2020), facilitating experimental design. Additional considerations in sgRNA design can further facilitate genome editing. First, targeting a sequence overlapping with a restriction site makes genotyping straightforward (Figure S8). Second, several reports suggest an improvement in Cas9 activity with sgRNA designs terminating in G-3′ or GG-3’ (Doench et al., 2014; Farboud and Meyer, 2015). Finally, in this article, a single experimenter was able to inject hundreds of eggs less than 60 min after egg-laying (AEL) in 2–3 h injection sessions. It is noteworthy that two experimenters, one collecting and aligning eggs, and the other injecting and sealing eggs, can inject eggs within 25 min AEL, which should reduce mosaicism and further increase germline transmission rates if needed.

Our CRISPR dominant rescue of the recessive white *c*.*737delC* mutation revealed efficient HDR-based single-base pair edits, showing ssODN donors can be routinely used to introduce small scarless edits in the *Plodia* genome. Asymmetric designs using homology arms ranging from 30 nt to 90 nt are generally recommended for such applications (Yoshimi, 2016), and verification of the edits is necessary as the technique can lead to imperfect repairs (Boel, 2018). In this article, we based our donor template design based on previous optimizations to single-base substitution performed in mammalian cell lines (Okamoto et al., 2019), using an ssODN located at or near the 5′ side of PAM sequence, complementary to the sgRNA strand, and around 75–85 nt in length with 30–40 nt of homology on both sides. Following these findings, our 76 nt repair template was designed to have a 40 nt left homology arm and 33 nt right homology with the nontarget strand (Figure 5A), a point mutation (C insertion) 4 nt away from the cutting site, and a 2 nt silent mutation (CTC > TTG Leu codon) to prevent secondary cutting. Because the WT (rescued) allele is dominant, we used a backcross to *w*^*-/-*^ and counted the number of G_1_ rescued individuals, as a proxy for estimating the proportion of germline-editing among mosaic founders. Backcrossing 10 of the 37.5% of G_0_ individuals in which we could detect somatic repair in the eyes, 24.4% of 496 G_1_ progeny showed rescued alleles from precise HDR. Injections for this experiment took place at 15–60 min AEL. As mentioned above, two experimenters injecting within 25 min AEL may limit both somatic and germline mosaicism and boost the editing rates. The dominant rescue assays suggest CRISPR methods enable precise germline editing in *Plodia* with relatively high efficiency, and we encourage the use and development of similar methods for targeted genetic transformation in this system.

### Limitations of the study

Although our study presents an approach to robust CRISPR/Cas9 NHEJ and HDR method in *Plodia*, we limited our knock-in attempts to a short ssODN template. The impact of longer repair template insertion on knock-in efficiency is yet to be determined. Efficient insertion of larger fragments in insects have been reported using donor dsDNA template, including the use of long donor dsDNA plasmids for HDR (Gilles et al., 2015; Ma et al., 2014; Purusothaman et al., 2021), microhomology-mediated end joining in *Bombyx* (Nakade et al., 2014), and the homology-independent end-joining of plasmid cassettes (Bosch et al., 2020; Matsuoka et al., 2021). These approaches are promising avenues for optimizing larger cassette insertion in *Plodia* genome, the third lepidopteran organism after *Bombyx,* and *Plutella* where germline-heritable CRISPR HDR edits are reported (Takasu et al., 2016; Wang et al., 2020).

## STAR★Methods

### Key resources table

**Table:** 

REAGENT or RESOURCE	SOURCE	IDENTIFIER
**Chemicals, peptides, and recombinant proteins**		

O'GeneRuler 100 bp DNA Ladder	Thermo Scientific	SM1143
Agarose, TBE buffer, gel electrophoresis set-up	Any	N/A
GelRed	Biotium	41003
Proteinase K, 20 mg/μL	Promega	V3021
DNARelease Additive	Thermo Fisher Scientific	F-170S
DNARelease Dilution Buffer	Thermo Fisher Scientific	F-170S
*Nla*IV Restriction Enzyme	New England Biolabs	R0126S
ExoI	New England Biolabs	M0568
rSAP	New England Biolabs	M0371S
Cas9-NLS protein	QB3 Macrolab, UC Berkeley	
RNAse Cocktail Enzyme Mix	Thermo Fisher Scientific	AM2286

**Critical Commercial Assays**		

DNeasy Blood and Tissue Kit (50)	Qiagen	69504
Taq 2x Master Mix	New England Biolabs	M0270
Plastic Pestle and 1.5 mL Tube	Bel-Art	F19923-0000

**Deposited Data**		

*Pi_Fog (w*^*-/-*^; *w^+/+^)* and *Piw-* whole-genome resequencing	NCBI SRA	PRJNA610057

**Experimental models: Organisms/strains**		

*Pi_Dun* WT strain, Dundee (UK)	Laboratory of Mike Boots, UC Berkeley	Roberts et al. (2020)
*Piw-* mutant strain (USA)	Laboratory of Paul Shirk, USDA/ARS	Shirk (2021)
*Pi_Fog* hybrid strain, *w*^*-/-*^ and *w*^*+/+*^	This work	N/A

**Oligonucleotides: DNA primers**		

*Pi_wh5_F*: *CGAACATGGCGTATAACTCC*	Genewiz	N/A
*Pi_wh5_R*: *CCAGAACACAGTGATCGGC*	Genewiz	N/A
*Pi_wh11-12_F*: *ACCTGACCACTGGTTGAC*	Genewiz	N/A
*Pi_wh11-12_R*: *CCACCCATCATCATTCCTTT*	Genewiz	N/A

**Oligonucleotides: sgRNA and ssODN**		

*Pi_wh5_KO_sg*: *TCGTCACAGAACATGAGTGG*	Synthego	N/A
*Pi_wh5_HR_sg*: *GGCTCGTCACAGAACATGAG*	Synthego	N/A
*Pi_ssODN_wh5_76NT*: GAAGAGGCTGTCCTTCGC CAGTGAAGTCCTGACTGATCCCCCATTGATGTT CTGTGACGAGCCCACATCGGGACTG	Integrated DNA Technologies	N/A
*Pi_BLOS2_KO_sg*: *CGTGGCAAGTCTGCTGATGA*	Synthego	N/A

**Software and algorithms**		

Geneious R10	Geneious	N/A
Profile-Hidden Markov Model Analysis (HMMER)	European Bioinformatics Institute	N/A
SWISS-MODEL	Biozentrum, University of Basel	N/A
Integrative Genomics Viewer (IGV)	Broad Institute	N/A

**Other: rearing containers**		

LocknLock Rectangular, 350 mL	LocknLock	HPL806
Copper Wire Mesh,100 × 100 Mesh, 0.0045″ Diameter Wire	Small Parts	CU-100-0045-01
Mason Jar, 16 oz	Ball	52150000
Steel Woven Wire Cloth Disc, 40 × 40 Mesh, 2–9/16″ Diameter	McMaster-Carr	2812T43
Stainless Steel Cup Holder (egg receptacle)	Da Vinci	B06W2JBLJJ
Cell Culture Flask with Vented Filter Cap, 25 cm^2^	SPL Life Sciences	70025
Plastic Souffle Cups, 1.25 oz	Dart Solo	T125-0090
Lids for 1.25 oz Cups	Dart Solo	PL100N
Cup Tray, 30 Wells	Frontier Agricultural Sciences	9040

**Other: microinjection**		

Cell Culture Dish, 35 mm	Nest Scientific	706001
Borosilicate capillaries with filament	WorldPrecision Instruments	18100F-3
Gravity needle puller	Narishige International	PC-10
Three-axis MM33 right-handed manipulator	Drummond Scientific	3-000-024-R
Single pressure micro-injectors with footswitch	Tritech Research Inc.	MINJ-1
Pulse-length control module	Tritech Research Inc.	MINJ-2
Needle holder	Tritech Research Inc.	MINJ-4
Compressed air faucet adapter	Tritech Research Inc.	MINJ-38NPT14QC
Polyurethane tubing 1/4″ OD, 1/8″ ID	Tritech Research Inc.	TT-1-4OD
3-way Tee air splitters	Tritech Research Inc.	MINJ-3TQC
4-way Tee air splitters	Tritech Research Inc.	MINJ4TQC
Brass compression fittings	Tritech Research Inc.	MINJ-5
Compression fittings	Tritech Research Inc.	MINJ-6
Binocular stereomicroscopes with 10–25× magnification	Any	N/A

**Other: Genomic resources**		

*Pi_dt* genome assembly (*Pi_Dun* WT strain)	NCBI Assembly	GCA_900182495.1
*Pi_dt* (*Pi_Dun* WT strain) whole-genome sequencing reads	NCBI SRA	ERS363461
*Pi_Sav* genome assembly (WT strain, Ag100Pest Consortium)	NCBI Assembly	PRJNA555319

### Resource availability

#### Lead contact

Further information and requests for resources and reagents should be directed to Dr. Arnaud Martin (arnaud@gwu.edu).

#### Materials availability

The *Pi_Fog w*^*-/-*^ and *w*^*+/+*^ strains are available to other laboratories upon request to the Lead Contact, and are compliant for interstate movement within the continental USA without additional permits (USDA ruling APHIS-2008-0076-0093).

### Experimental model and subject details

*Plodia* cultures are maintained in dedicated containers at 28°C with an relative humidity of 60% and a 14:10 h light:dark cycle (see STAR Methods). The *Pi_Dun* strain, a kind gift of Mike Boots (Roberts et al., 2020), was used as a wild-type strain with a reference genome assembly (NCBI Assembly database: *Pi_dt*). The *Pi_Fog* strain consists of an introgression of the spontaneous *w^-^* mutation (Shirk, 2021), from the *Piw-* strain (origin: USA, a kind gift of Paul Shirk), into the genomic background of *Pi_Dun*. For 13 of the 15 generations of crossing (Figure 1A), pupae were sexed, transferred to individual plastic cups, mixed with an individual from the opposite sex after emergence, supplemented with diet after 3 days, and transferred to a 350 mL rearing container after confirmation of larval eclosure. About 6-8 crosses were performed at each generation, and *w*^*+/-*^ heterozygous genotypes were inferred from phenotype segregation among offspring. Two generations consisted of random matings within mass-rearing containers. At the end of this crossing scheme, *w*^*+/+*^ and *w*^*-/-*^ from the resulting hybrid *Pi_Fog* strain were isolated and both genotypes have been successfully maintained in inbred state for 2 years. More detailed methods for the use of *Plodia* as a laboratory model system for functional genetics are presented in the supplemental Information (Document S1).

### Method details

#### Whole-genome resequencing

Adult individuals with *Piw-*, *Pi_Fog w*^*-/-*^, and *Pi_Fog w*^*+/+*^ genotypes were isolated for whole-genome resequencing. For each individual, the head and thorax were placed in a 1.5 mL microtube, lyzed with a matching pestle in 180 μL Buffer ATL (Qiagen), incubated with 400 μg Proteinase K at 56°C for 20 min, and supplemented with 12 μL of RNAse Cocktail (Thermo Fisher Scientific) for 12′ at room temperature. DNA was extracted with the DNeasy Blood and Tissue kit (Qiagen), eluted using 70 μL of Buffer AE (Qiagen, 10 mM Tris-Cl, 0.5 mM EDTA, pH 9.0), and quantified using a Qubit 3 fluorometer and the HS dsDNA kit (Thermo Fisher Scientific), yielding between 0.7 and 4.2 μg of DNA per individual. A minimum of 1 μg per individual was used for preparation of five barcoded samples, which were sequenced in a large multiplexed library on a NovaSeq S1 150 bp PE run (Illumina). Raw reads are available on the NCBI SRA under BioProject accession number PRJNA610057.

#### Association mapping of the *w-*allele

Reads were aligned to the *Plodia* Savannah strain genome assembly using BWA-MEM with default parameters (Childers et al., 2021; Li, 2013). Variant calling was performed in GATK using tools HaplotypeCaller and GenotypeGVCFs with default parameters, with the output filtered for just bi-allelic SNPs using Samtools (Li et al., 2009; McKenna et al., 2010). Population statistics were plotted using code from Simon H Martin (2021), with *F*_*ST*_ calculated in 100 kb windows at 10 kb sliding intervals. Chromosome genotype plots were generated using the R package GenotypePlot (Whiting, 2021). Variant call files (VCFs) for the genes *white*, *cinnabar* and *scarlet* were examined in IGV.

#### *In-silico* protein analysis

The reference genome sequence (NCBI Assembly *Plodia_dt*: the “Dovetail” version of the WT - Dundee strain *Pi_Dun*) was used to identify a complete coding sequence, and was translated into a 685 amino acid residues. White protein sequence. Protein family domains were visualized using profile-hidden Markov modeling, HMMER web server 2.41.1 (Potter et al., 2018) following a search against the *Drosophila melanogaster* Reference Proteome in UniProt. To visualize the tertiary and quaternary structure of the White protein, we generated the 3D model of the protein using SWISS-MODEL (Waterhouse et al., 2018). The protein was categorized as ABCG2 and was fitted into the available 6hbu.1.A human ABCG2 mutant protein template (Manolaridis et al., 2018). The generated template offered 0.87 coverage with tertiary and quaternary structure quality estimate scores (GMQE and QSQE) of 0.62 and 0.47.

#### Detailed rearing procedure

*Plodia* is well suited for laboratory culture, and routine stock maintenance involves a single human intervention per generation. Briefly, adult moths are left to mate in a mass-rearing container and anesthetized with CO_2_, which induces oviposition and allows the collection of large quantities of eggs (Figures S4A–S4G). As fertilization occurs at oviposition (Lum et al., 1981), this generates synchronized embryos that can then be used for inoculation of a new stock (Figures S4H–S4J), or for microinjection (Figures S4K and S4L).

A wheat bran artificial diet suitable for optimal larval growth was adapted from a previously published protocol (Silhacek and Miller, 1972). Following mixing of the ingredients (Table S1), the diet can be kept refrigerated in a tupperware container for up to two months before use, after which its drying can severely impact the insect cultures.

Larval containers must be completely insect proof, tolerate freezing temperatures for devitalization, and resist accidental falls without opening. We recommend food storage containers with tight-locking lids and thick walls (e.g. LocknLock Rectangular, 350 mL). Vents must be added to allow air circulation: a 2 cm diameter hole is perforated in the lid, and 100 × 100 copper wire mesh is used to cover the hole on the inside of the lid, with a soldering iron used to melt the mesh into the plastic without (Figure S5).

*Plodia* adults of the *Pi_Fog* strain emerge about 25 days after fertilization when reared at 28°C. Moths containers are then left one to two days before egg collection to ensure fertile matings. To pass the stock, the container is saturated with CO_2_ gas until all adults are anesthetized, then the *Plodia* adults are quickly transferred into a mason jar covered with a steel 40 × 40 mesh disc under the lid (oviposition jar, Figure S6). CO_2_ narcosis induces oviposition, resulting in a rapid accumulation of fertilized eggs within 2 h. Silanization of the inside of the container (e.g. Rain-X glass treatment) can help reduce the sticking of eggs to the jar, but it is usually not required. For inoculation of a new generation, eggs are transferred to a stainless steel cup (which avoids static charging), by inversion and tapping of the eggs through the mesh lid of the oviposition jar. Eggs are weighed on a weighing boat, which is then placed inside a rearing container with compressed diet. For 350 mL containers, this is dones at a ratio of 10–12 mg of eggs for 50 g of diet. A piece of filter paper is taped over the mesh vent (Figure S4A) to prevent dessication. The stock can then be left unattended for the next 25 days (peak of adult emergence), although we recommend periodic monitoring of

#### Embryo microinjections

To anesthetize and induce synchronized egg-laying, adult *Plodia* moths in a rearing container are exposed to CO_2_, then transferred to the oviposition jar. The jar is tapped upside down to remove older eggs. After 10–12 min of oviposition at room temperature, the eggs are collected in a metal container (Figure S7A). Using a wet paintbrush, the eggs are picked up and arranged on a 1 × 3 cm parafilm in a cell culture dish (Figure S7B) such that the micropyle is on the opposite side of the intended injection site (Figure S7C), adding a droplet of water as adhesive on the bottom, if necessary. Using a microcapillary needle, a droplet of injection mix is pushed by air pressure into the posterior side of the egg (Figure S7D). After injection, it is important to seal the site of injection by tapping onto the top of the egg a fine copper wire dipped in thin glue (Figure S7E). The time from egg collection to egg sealing is kept under 60 min. The total injected eggs are then counted and incubated at 28°C. For the first 24–48 h, injected eggs are kept in the rearing container in the presence of a slightly damp tissue wipe, and with tape over the copper mesh hole. This procedure provides humidity saturation that prevents the eggs from drying out. After 24–48 h, the tape is removed to allow ventilation, and the wet tissue wipe is removed from the container, preventing molding. To prevent the escape of larvae outside of the injection dish, it is recommended to add a small amount of artificial diet (one or two flakes per dish) when removing the wipe tissue, as this will attract hatchlings and facilitates counting and transfer in further procedures.

#### CRISPR NHEJ knock-outs

Syncytial embryos of *Pi_Fog w*^*+/+*^ were injected with Cas9 and short guide RNA (sgRNA, *Pi_wh5_KO_sg* or *Pi_BLOS2_KO_sg*) complexes at 500:250 ng/μL concentration, within 15–60 min after egg laying (AEL). Pupae were then individually isolated from cardboard “hotels” for phenotyping under dissection microscope and outcrossing (Figure S6). Selected *white exon 5* mutants lacking any pigmentation in both eyes were crossed with the *w*^*-/-*^ strain to determine the germline transmission of NHEJ-induced null mutations.

#### CRISPR HDR rescue

To investigate whether the insertion of cytosine at position 737 in *white* exon 5 is necessary to restore the brown eye phenotype in the *Pi_Fog w*^*-/-*^ strain, eggs were injected with Cas9, *Pi_wh5_HR_sg* RNA, and *Pi_ssODN_wh5_76NT* single-stranded oligo donor (ssODN) complex at 500, 250, and 1000 ng/μL concentration, respectively. All eggs were injected within 15–60 min AEL.

#### Isolating virgins for controlled crosses

At the end of the wandering larval stage (L5), when provided with cardboard “hotels” (Figure S8), larvae enter and spin silk to plug the openings of the cardboard. Hardened pupae are extracted from the thin cocoon and lined up on a double-sided tape that has been dabbed with a Kimwipe to reduce stickiness. The courtship behavior of male and female *Plodia* (Grant et al., 1975) consists of a stereotypical sequence initiated with the pheromone call of females that show an upward-bending of the abdomen to expose a posterior gland. If males are sexually mature (in our experience, at least 24 h after emergence), they often immediately respond to this call and perform a rapid fanning of their wings to spread wing pheromones while walking towards the female, followed by copulation. Successful matings are easily visualized at the bench, often seconds after mixing of virgin males and females in the same container. If mating does not successfully occur, it can be elicited by placing the container in the dark, or it may indicate that the individuals have emerged too recently.

#### Genotyping assays

For Restriction fragment-length polymorphism (RFLP), one leg of *Plodia* was submerged into 0.5 μL DNARelease Additive and 20 μL Dilution Buffer, heated at 98°C for 2 min, and 1 μL of this lysate was used for PCR in a 15 μL volume (95°C for 5 min, followed by 30 cycles with 95°C for 15 s, 56°C for 15 s, 68°C for 60 s; and final elongation at 68°C for 1 min) using 7.5 μL Taq 2x Master Mix and 0.2 μL of each primer (10 μM stock). Alternatively, a pupa can be pricked between dorsal thoracic segments with a tungsten needle for sampling ∼0.2 μL of hemolymph, which then is mixed with 10 μL of the DNArelease/Dilution buffer solution. A volume of 2 μL of this DNA extract can then be used for PCR amplification.

For the genotyping of *c.737delC* the 40 μg of product amplified by *Pi_wh11-12_F/R* primers was digested for 1 h at 37°C with 1 U of *Nla*IV (New England Biolabs). For restriction analysis, 5 μL of the restriction product and 0.5 μL O’GeneRuler 100 bp DNA ladder (Thermo Fisher Scientific) were loaded onto a 2% agarose gel with 0.5 X TBE buffer and electrophoresed at 50 V (Figure S9).

For Sanger sequencing, 40 ng of PCR product was purified with 1 U ExoI and 1 U of rSAP (New England Biolabs) in a 6.5 μL reaction volume. The reaction was incubated for 15 min at 37°C followed by enzyme inactivation for 15 min at 80°C. Then, 25 pmol of *Pi_wh11-12_R* primer was added to 3.2 μL of purified PCR product in 15 μL total volume per reaction.

#### Biosafety measures for wild-type stock

*Plodia* pantry moths are non-infectious, non-parasitic, non-vector, and non-exotic insects that are found on all continents except Antarctica, and generally considered as native pests of stored food products. They are notorious for their infestation potential in kitchens and pantries, and can proliferate within a laboratory building if baseline containment measures are not taken. We prescribe important recommendations for working with this insect at the wild-type state.

• *Transfers between primary containers.* The entire lifecycle is maintained in escape-proof containers, so the risk of egg/diet spills or flying moth escapes is limited to moments where these containers are opened. The transfer of narcosed adults to an oviposition jar should be quick, over a tray (Figures 4B and 4C), and under a fumehood. Lab surfaces must be wiped with ethanol if potentially contaminated with eggs. The transfer of adults isolated in cups or flasks must be done inside a secondary container, such as a mesh cage.

• *Secondary containment. Plodia* cultures should be in a dedicated incubator or in a clean insectary with limited access and no direct escape routes. “Pantry moth” pheromone traps are highly effective for detecting an early infestation, and may help to catch accidentally released males before they mate. Indoor ultraviolet insect traps can also be installed as preventive measures in the insectary and adjacent rooms.
• *Lab environment.* Food must be prohibited in the laboratory areas surrounding the insectary. In addition, *Plodia* can infest the wax of laboratory bee cultures.
• *Disposal and cleaning.* Stringent measures to devitalize all the material for disposal in order to avoid a laboratory infestation. All containers, individuals (including excess eggs), cultures and waste (wipes) are sealed in a plastic bag and frozen at −20°C for at least 72 h in order to achieve complete disinfestation, a conservative procedure based on published measures of an LT_95_ estimate of 7 h at this temperature (Johnson, 2007). Dilute bleach (5%) treatment for 10 min dissolves egg chorions and is an effective chemical decontaminant for surfaces and equipment that can not be frozen.
• *Personal protective equipment.* Disposable gloves are recommended to avoid contaminating cultures with mold. Lab coats are recommended to avoid the export of egg material outside of the lab. Most importantly, experimenters must use respiratory protection (fumehood, respiratory filter) when opening or cleaning mass-rearing containers, due to the allergen potential of moth scale volatiles (Binder et al., 2001).

#### Arthropod containment level 2 measures for genetically modified stock

The generation and maintenance of transgenic or genome edited arthropod stock is prone to formal regulations that are country or institution specific. Published guidelines about Arthropod Containment Levels 1 and 2 (ACL-1 and ACL-2) protocols (American Committee of Medical Entomology-American Society of Tropical Medicine and Hygiene, 2019) provide an advisory framework for safe genetic modification in *Plodia*. While the ACL-2 standard was designed for arthropods of public health concern, it is flexible and overall a good target for responsible research conduct, but may be insufficient if the laboratory modifications present a risk of fitness advantage or genetic contamination in the wild (*e.g.* insecticide resistance, gene drives). In addition to the aforementioned baseline recommendations with wild-type *Plodia* stock, we advise the following when working with genetically modified *Plodia*.

• *Disposal.* All material must be treated as Biohazardous waste (similar to transgenic *Drosophila*) following ACL-2 protocols.
• *Facilities.* Simple ACL-2 barriers to flying and crawling insects, for instance using a dedicated insectary or incubator room with self-closing doors, and custom ventilation meshes, should be accommodated whenever possible. More expensive facilities with double-door vestibules, or negative air pressure, are not needed for *Plodia,* since accidentally released adult moths should be extremely rare with the aforementioned primary containment procedures.
• *Personel.* ACL-2 measures have increased stringency on access and training compared to ACL-1, which are important to reduce the risk of accidental releases due to negligence.

### Quantification and statistical analysis

Our study did not include statistical analysis or quantification.

## Data Availability

All short-read data generated in this project are available on the NCBI repository under accession number PRJNA610057. This study did not generate new code.
