# Mapping and CRISPR homology-directed repair of a recessive white eye mutation in *Plodia* moths

**DOI:** 10.1016/j.isci.2022.104749

**Published:** 2022-07-18

**Authors:** Christa Heryanto, Joseph J. Hanly, Anyi Mazo-Vargas, Amruta Tendolkar, Arnaud Martin

## Main text

(iScience *25*, 103885; March 18, 2022)

In Table S1 (related to the STAR Methods) the quantities of some of the ingredients of the *Plodia* artificial diet were incorrect. The correct amount of “Brewer’s yeast flakes” is 90 g but was originally erroneously reported as 121 g, and the correct amount of “glycerin, USP grade” is 230 g but was originally reported as 197 g. The authors apologize for this error. The table has been updated in the original article.

