# Supplementary material for: Mapping and CRISPR homology-directed repair of a recessive white eye mutation in Plodia moths
Source: iScience. 2022 Feb 5;25(3):103885. doi: 10.1016/j.isci.2022.103885 (PMC8861637; doi:10.1016/j.isci.2022.103885)
Supplement: Document S1. Figures S1–S9 and Table S1 [file mmc1.pdf]

iScience, Volume *25*

Supplemental information

Mapping and CRISPR homology-directed repair

# of a recessive white eye mutation in *Plodia* moths

Christa Heryanto, Joseph J. Hanly, Anyi Mazo-Vargas, Amruta Tendolkar, and Arnaud Martin

SUPPLEMENTAL INFORMATION

This Document S1 contains Table S1 and Figures S1-S9

Table S1. *Plodia* artificial diet, Related to STAR Methods.

Ingredient	Source / Identifier	% (w/w)	Weight (g)
Coarse wheat bran, organic	Shiloh Farms / 47593202022	50%	454 (1 lb)
Brewer’s yeast flakes	Kal / B00020HV1E	13%	121
Dextrose, anhydrous	Amresco / 0188-500G	5%	45
Glycerin, USP grade	Bluewater / 53131607	22%	197
Canola oil	-	2%	15
Water	-	8%	76

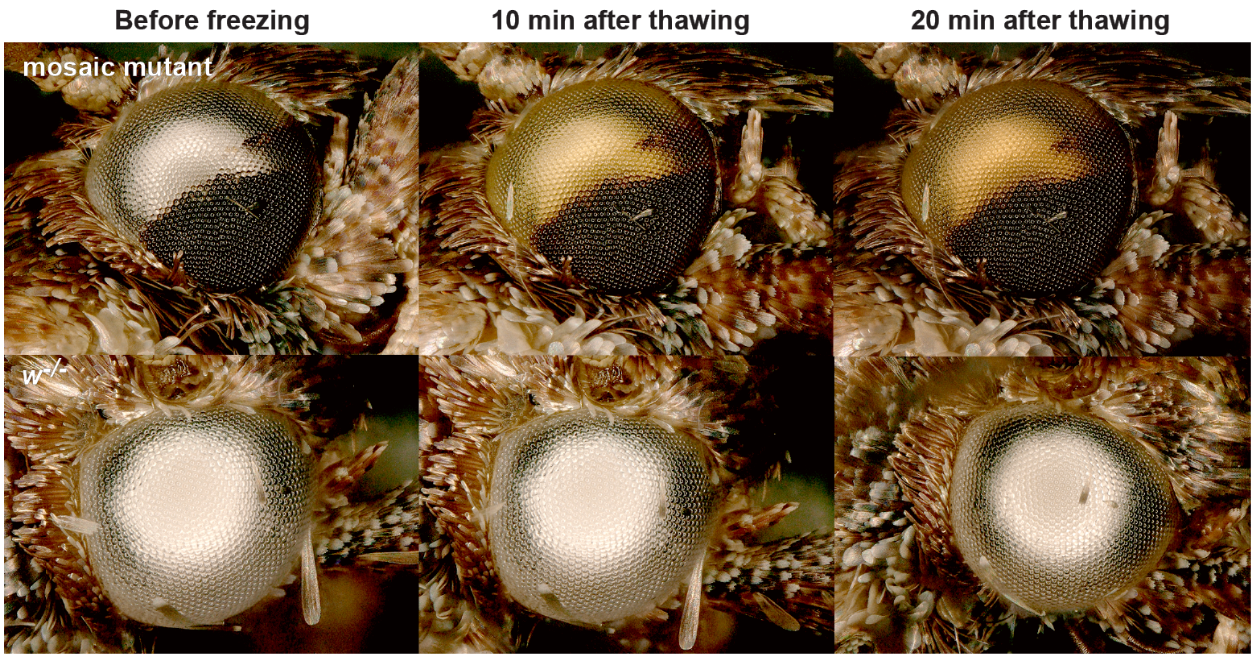

Figure S1. Thawing results in artifactual pigment diffusion in mosaic *white* mutant eyes, Related to Figure 4. Pigment diffusion into the white area of the eye is observed within 20 min post thawing in mosaic eye mutants. $w^{-/-}$ control individuals remain unchanged.

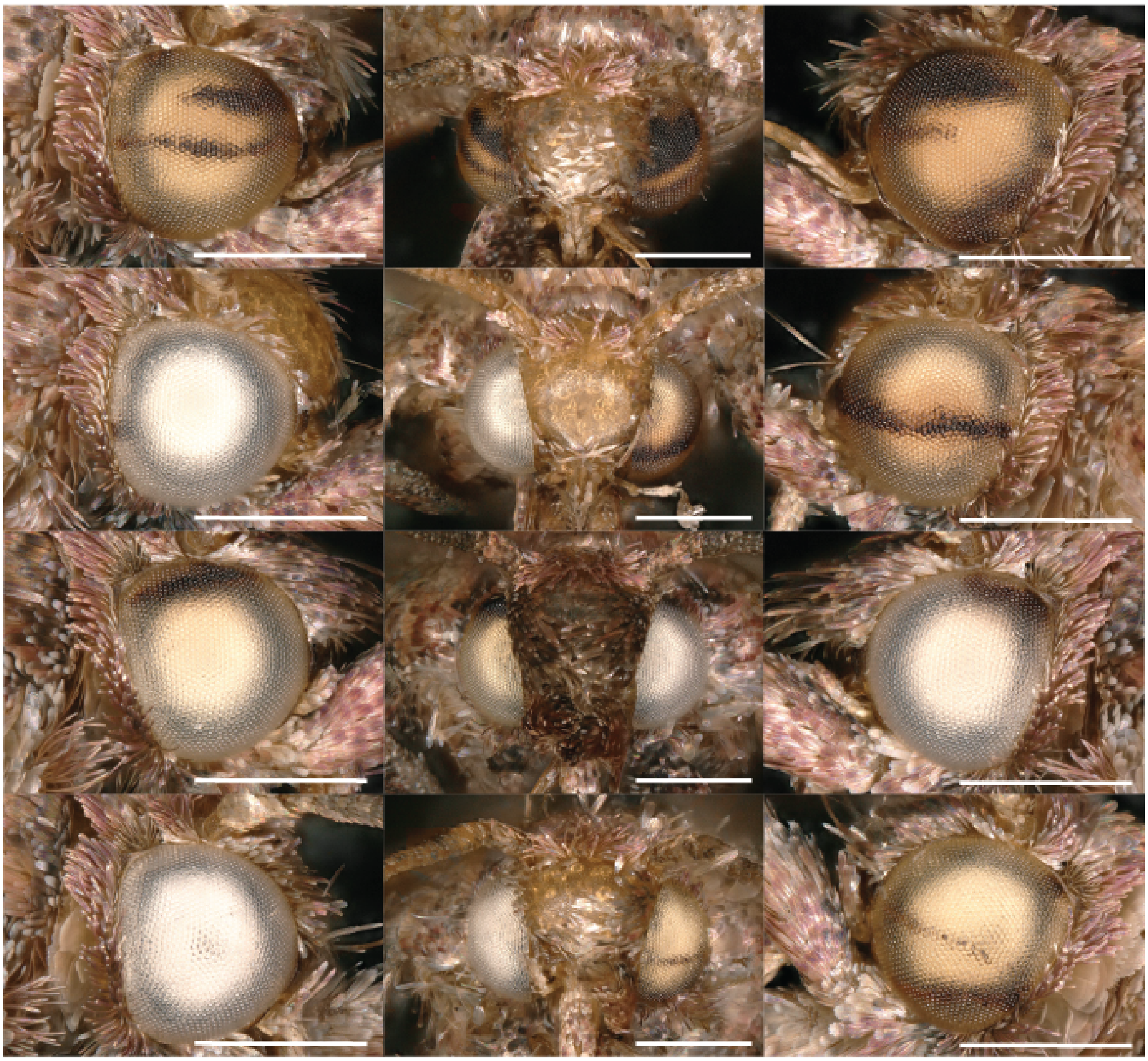

Figure S2. G_0 eye color phenotypes generated by HDR-induced *white* rescue in mutant *Plodia*, Related to Figure 5*.* Examples of G_0 phenotypes featuring small HDR rescue clones following CRISPR/ssODN injection in white-eyed *Pi* $w^{-/-}$. Scale bars = 500 µm.

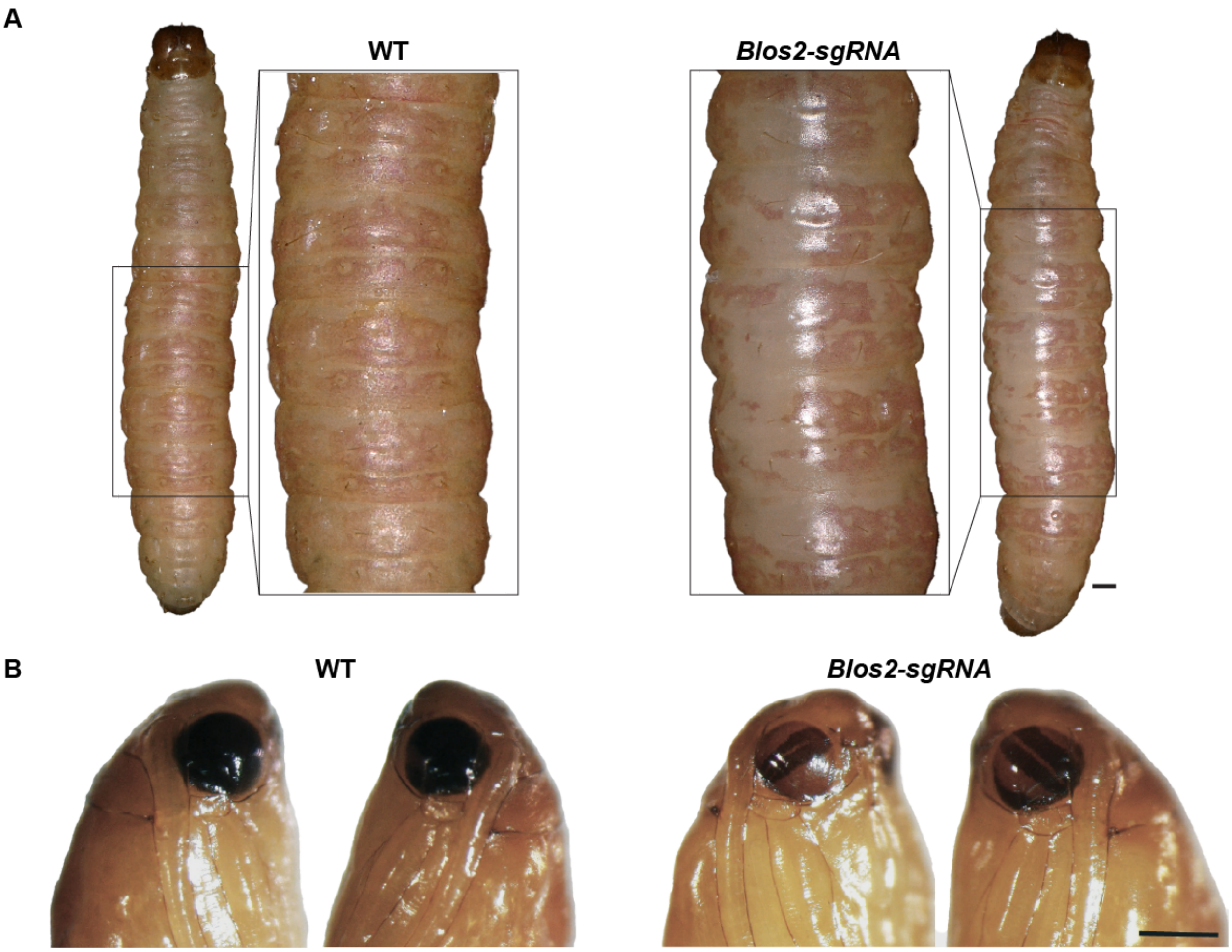

Figure S3. G_0 color phenotypes in larvae and pupae following CRISPR gene knockout of *Blos2*, Related to STAR Methods. (A) Disruption of the pink integument pigmentation of fifth instar larvae compared to wild-type controls. (B) Eye discoloration phenotypes detectable at intermediate pupal stages (20-60% pupal development). Mutant clones were only visible before final eye melanisation subsequent to the P8 stage (Zimowska et al., 1991). Scale bars = 500 μm.

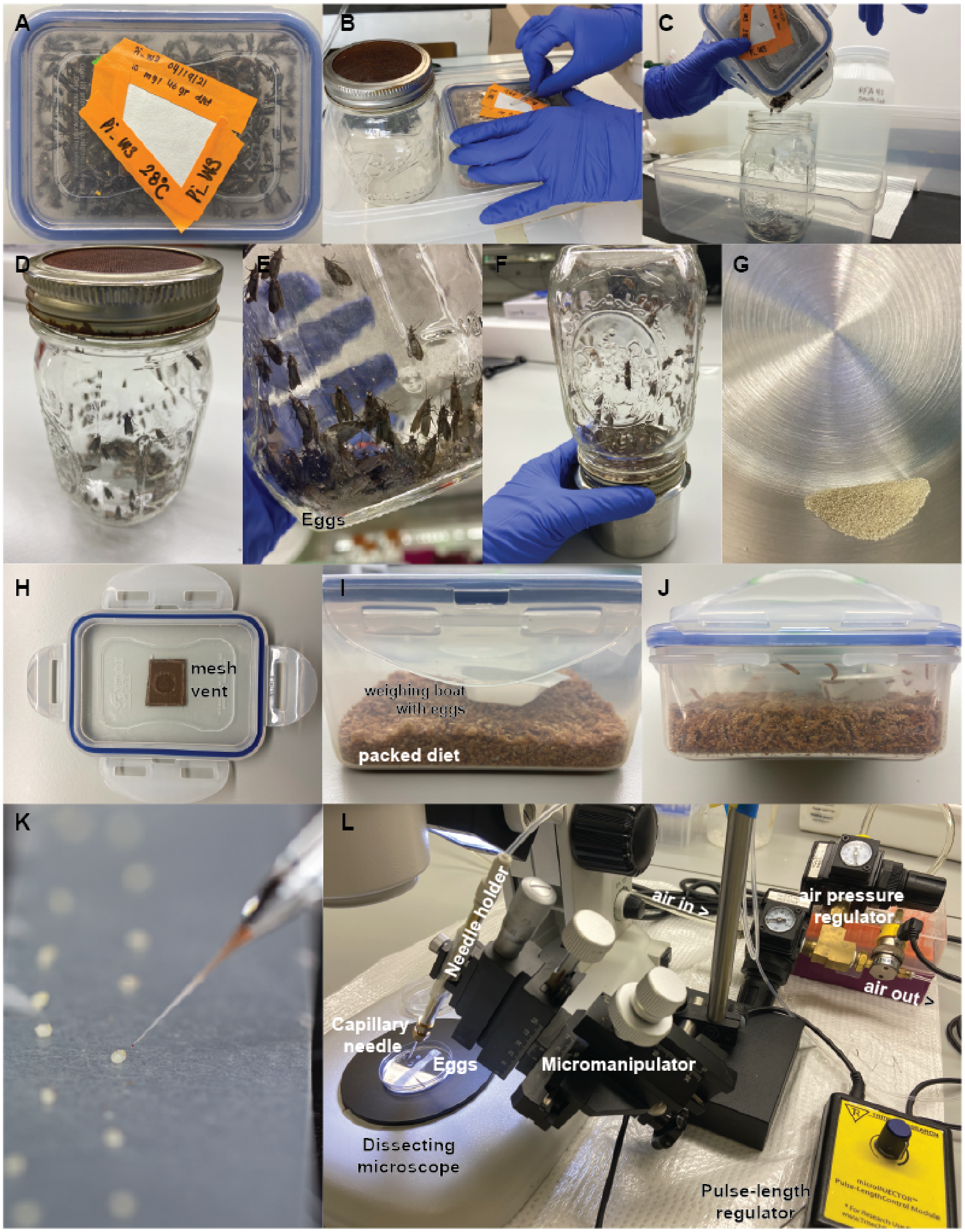

Figure S4. Overview of lab rearing and injection setup for *Plodia* microinjections, Related to STAR Methods. (A) Mated *Plodia* adults in a mass-rearing container. (B) Injection of CO_2 through the container vent. (C) Transfer into an oviposition jar. (D-G) Egg collection in a metal cup. (H-J) Inoculation of synchronized embryos in a vented, escape proof container. (J) Stock at the fifth instar larval stage. Wandering larvae outside of the diet are looking for a pupation site. (K) Egg injection with microcapillary needle carrying CRISPR reagents. (L) Microinjection setup.

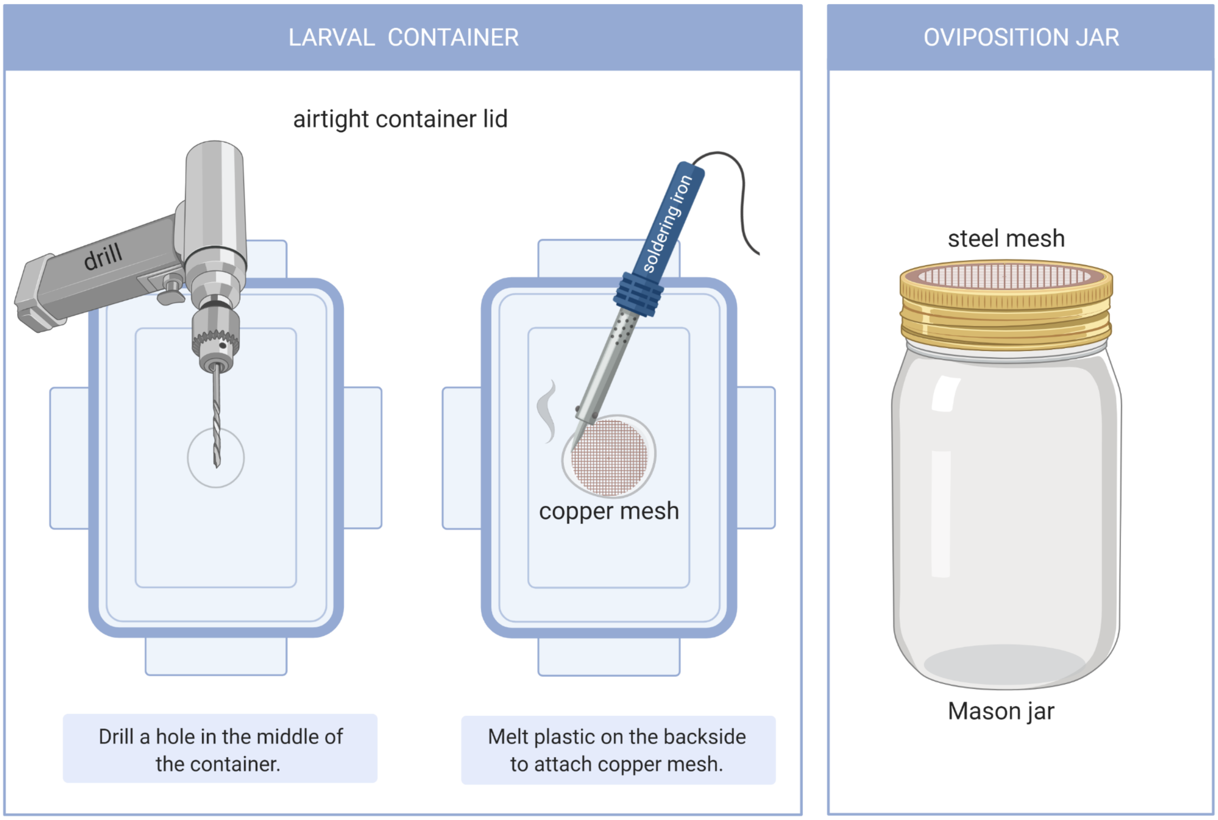

Figure S5. *Plodia* rearing and oviposition containers, Related to STAR Methods.
Left : procedure for the modification of larval container lids. A drill fit with a spade bit is used to make a 2 cm wide diameter hole, and soldering iron is used for melting copper mesh on the inner side. Right: Oviposition jar is made by replacing the removable lid of the glass Mason jar with a steel mesh disk. This mesh allows egg collection when the jar is inverted while keeping adult *Plodia* moths in the jar.

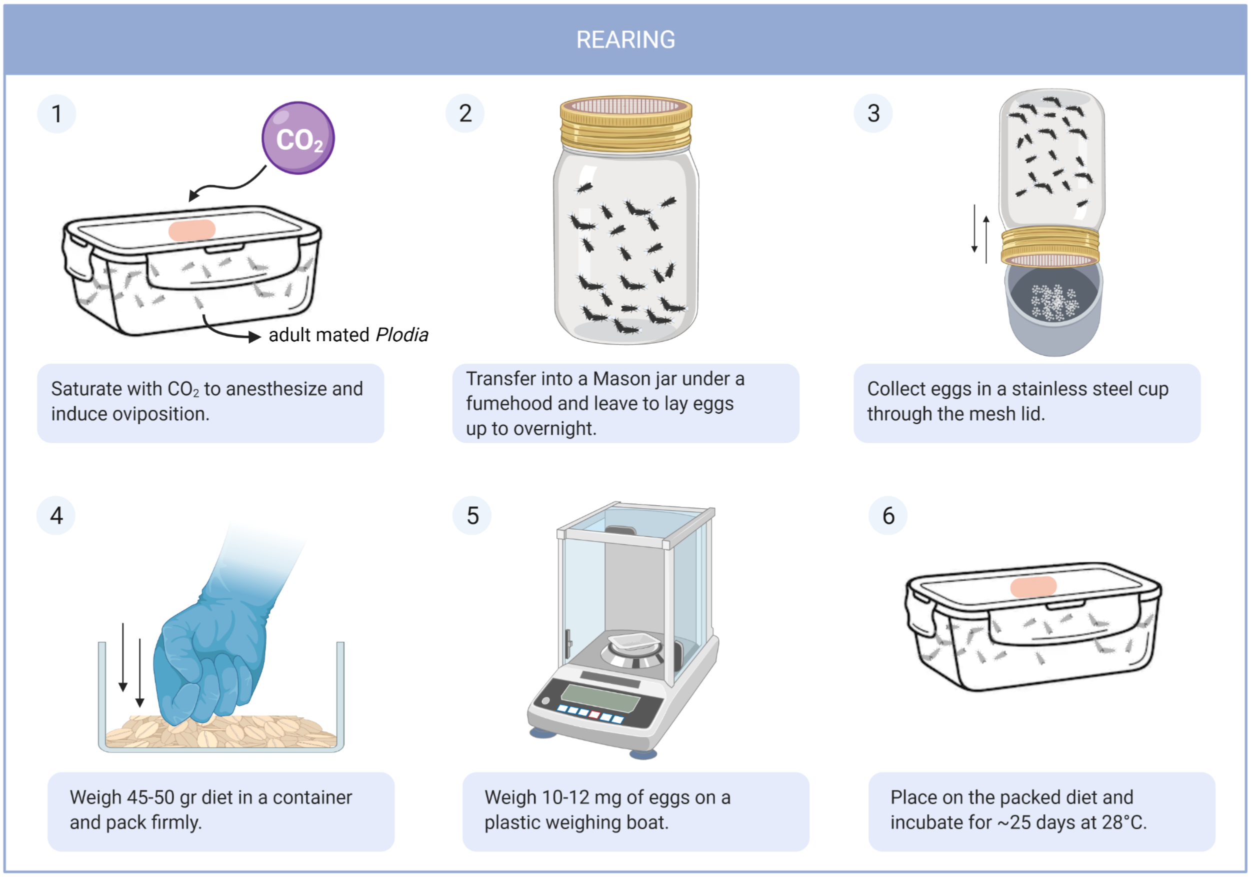

Figure S6. Inoculation of a standard *Plodia* culture, Related to STAR Methods.

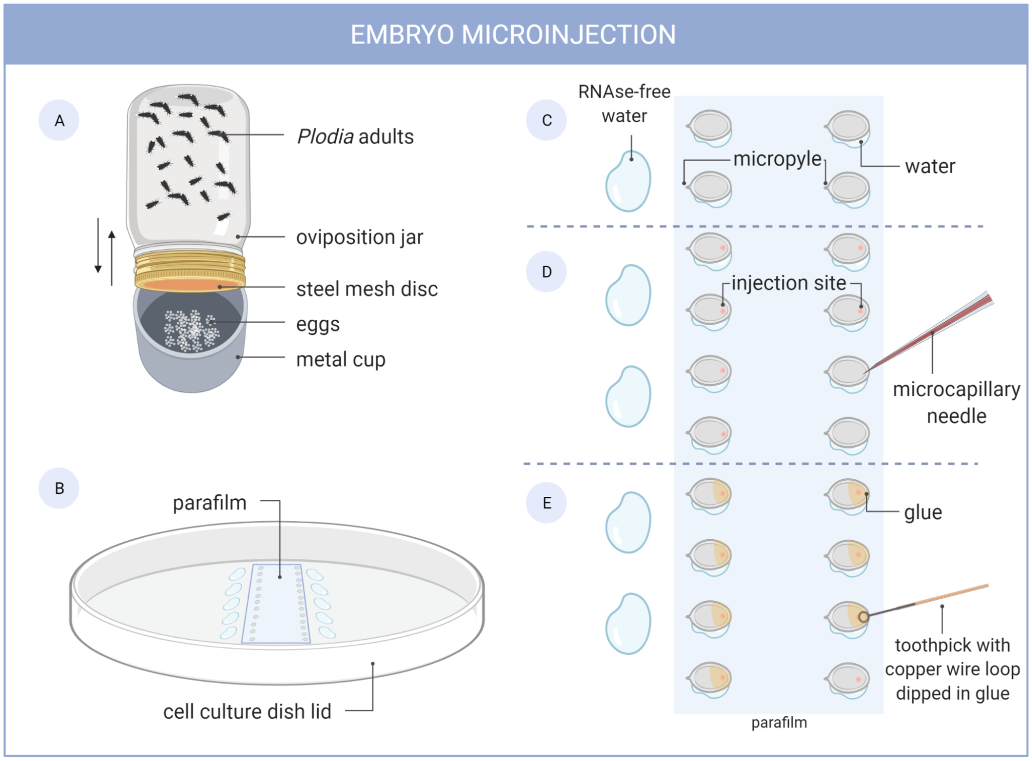

Figure S7. Summary of the embryo microinjection workflow, Related to STAR Methods.
(A) Egg collection from an oviposition jar. (B) Injection pad using cell culture dish lid. (C) Egg orientation on parafilm. (D) Microinjection with glass needle. (E) Sealing with glue.

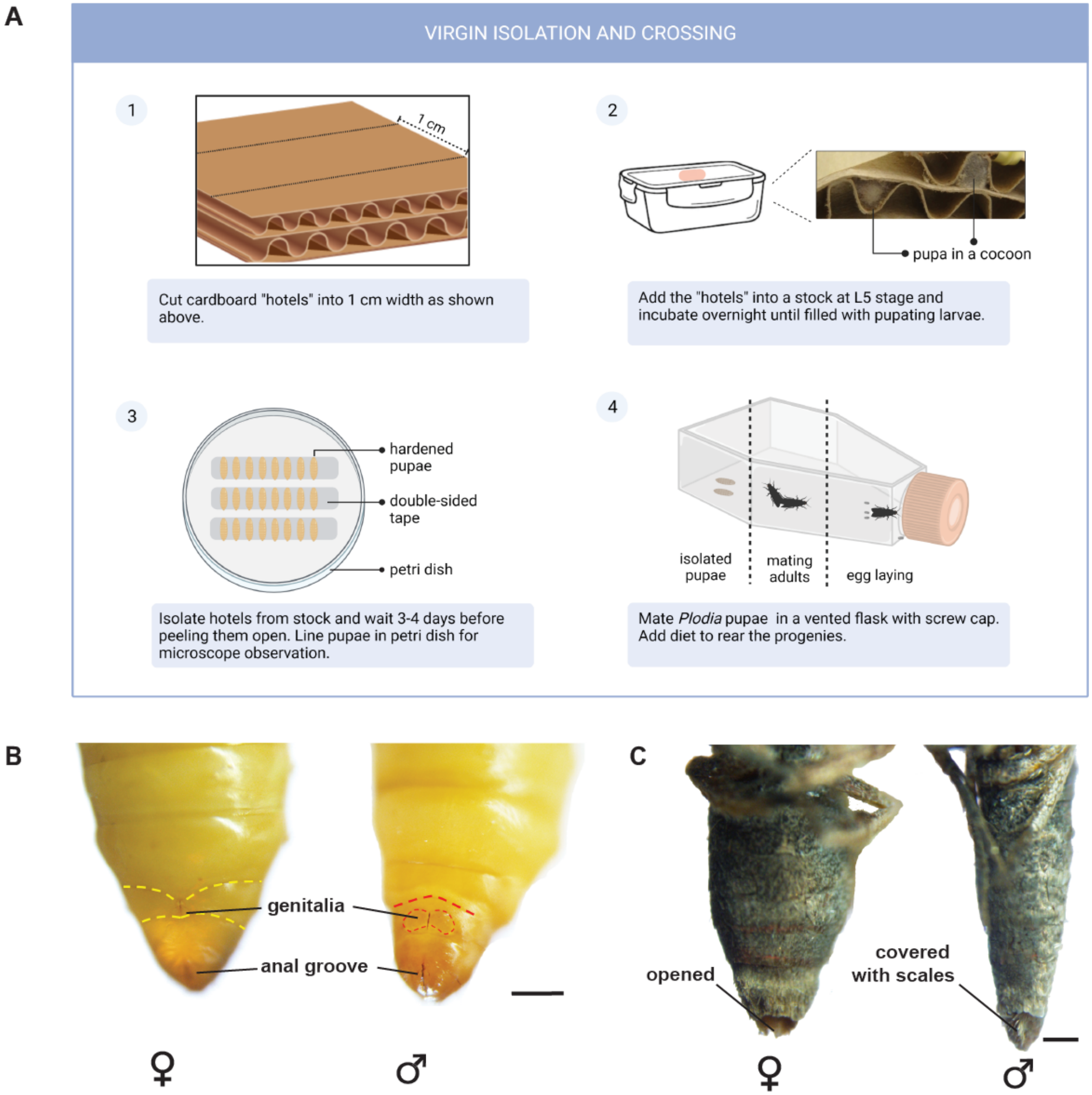

Figure S8. *Plodia* sexing and crossing, Related to STAR Methods.
(A) Unmated isolation and crossing procedure. (B) Sexing *Plodia* pupae. In females, the abdominal segments on the ventral side near their genitalia are shaped distinctively (yellow dotted lines) from males. Males have a pair of rounded sex marker near their genitalia (red circle dotted lines). The distance between anus and genitalia in male is generally shorter than in female pupae. (C) Sexing *Plodia* adults. The difference in shape of the posterior end of female and male adults. Female adults tend to have rounder abdomens and larger bodies due to the eggs they are carrying.

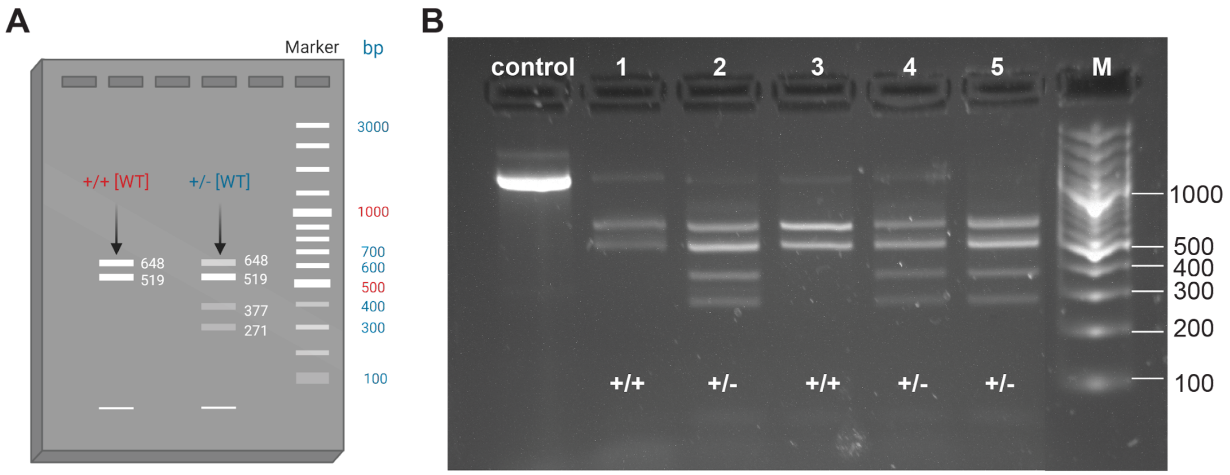

Figure S9. RFLP genotyping of the recessive *w-* allele in wild-type moths, Related to STAR Methods.
(A) Expected bands for *Plodia* genotyping using *white* amplicon digested with the *Nla*IV restriction enzyme distinguishing homozygous (+/+ [WT]) and heterozygous (+/-[WT]) samples. (B) Gel electrophoresis image. Control: negative control of undigested *white* amplicon at 1,211 bp. Lane 1, 3: digested $w^{+/+}$ with 519 and 648 bp bands. Lane 2, 4, 5: digested $w^{+/-}$ with 271, 377, 519, and 648 bp bands. M: 100 bp DNA Ladder.
